# Primary Hyperparathyroidism: Experience from a Tertiary Care Centre in Pakistan

**DOI:** 10.12669/pjms.36.6.2572

**Published:** 2020

**Authors:** Tehseen Fatima, Bhagwan Das, Saadia Sattar, Najmul Islam

**Affiliations:** 1 Dr. Tehseen Fatima, FCPS. Section of Endocrinology, Department of Medicine, Aga Khan University Hospital, Karachi, Pakistan; 2 Dr. Bhagwan Das, FCPS. Section of Endocrinology, Department of Medicine, Aga Khan University Hospital, Karachi, Pakistan; 3 Saadia Sattar, MSc. Section of Endocrinology, Department of Medicine, Aga Khan University Hospital, Karachi, Pakistan; 4 Dr. Najmul Islam, FRCP. Section of Endocrinology, Department of Medicine, Aga Khan University Hospital, Karachi, Pakistan

**Keywords:** Primary Hyperparathyroidism, Hypercalcemia, Osteitis fibrosa cystica

## Abstract

**Objective:**

To study the clinical, biochemical and radiological features and management outcomes of patients with primary hyperparathyroidism.

**Methods:**

This retrospective study was conducted at the Aga Khan University Hospital, Karachi, Pakistan and comprised data of patients with primary hyperparathyroidism from 2008 to 2017.

**Results:**

Out of 103 patients, 83(80.6%) were female. Overall mean age was 59.3±16.2 years. Musculoskeletal manifestations were seen in 60(58.3%) patients and renal manifestations in 28(27.2%). Ostieits fibrosa cystica was found in 04(3.88%) patients. Overall, Ultrasound neck and sestamibi scan localized the lesion in 66 (64.1%) and 77 (76.2%) patients respectively. Among 79 patients who underwent surgery, 67 (84.8%) patients had an adenoma, 05 (6.3%) had hyperplasia and 02(2.53%) patients had parathyroid carcinoma whereas histopathology was inconclusive in 5 (6.32%) out of the 79 surgically treated patients. Disease recurrence was seen in 13 out of 79(16.45%) patients who underwent surgery.

**Conclusion:**

Primary hyperparathyroidism is associated with significant morbidity in our population. Targeted measures like improving patient awareness, routine calcium screening, vitamin D supplementation and a high index of suspicion by the clinician may help in early diagnosis of the condition and thus reduce morbidity.

## INTRODUCTION

The prevalence and clinical presentation of Primary Hyperparathyroidism (PHPT) is strikingly different among populations.^1^-^4^ The difference is even more pronounced between the West and the East. With the introduction of routine calcium screening in the Western world, the phenotype there has largely shifted from symptomatic to asymptomatic and being detected at early stages.^5^ Contrary to this, in the developing countries, where there is no routine screening of calcium, PHPT continues to present as a symptomatic disease.^6^-^9^ Data from the Rochester Epidemiological Project reported the incidence of PHPT as 21.6 cases per 100000 person years.^1^ There are very few studies regarding PHPT in Pakistan and the exact prevalence in not known.^8^-^10^ This study was designed to analyze the clinical presentation, complications, and management outcomes in patients with PHPT in order to have a better understanding of the disease spectrum in Pakistan.

## METHODS

This retrospective study was done after EC/IRB approval (2019-0850-2587, Dated: 9 February 2019) at the Aga Khan University Hospital, a tertiary Care Centre in Pakistan. This hospital is located in Karachi and mainly caters to an urban population. Data was collected from the medical records of patients with PHPT from January 2008 to December 2017. This included patient demographics, clinical presentation, laboratory parameters such as Serum PTH and calcium at presentation and on follow up after treatment, serum phosphate, vitamin D, albumin, imaging, treatment opted and histology. All calcium values were corrected for respective albumin concentrations. Diagnosis of PHPT was made on the basis of hypercalcemia or normocalcemia with high or inappropriately normal serum PTH levels. All patients aged 18 years and above with PHPT were included. Patients with renal failure and hypercalcemia due to malignancy were excluded.

All clinical and radiological features and management outcome of patients were reported as frequency and percentages while biochemical parameters were reported as mean with Standard deviation (depending on the normality assumption) or Median with Interquartile range (if normality assumption was not met). Stata version 12 was used for analysis.

## RESULTS

**Fig.1:**
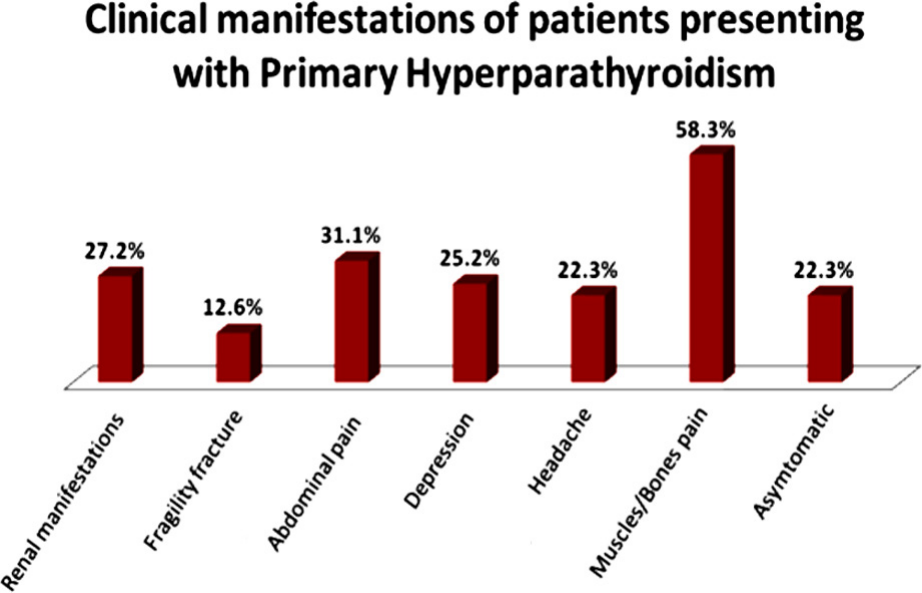
Clinical manifestations of patients presenting with primary hyperparathyroidism.

A total of 103 patients were identified with PHPT from 1st Jan, 2008 till 31st Dec, 2017 with mean age of 59.3±16.2 years. Majority of the patients were female [83 (80.6%)]. Patients presented with various clinical manifestations such as 60(58.3%) with muscle and/or bone pain, 32(31.1%) with abdominal pain and 28(27.2%) with renal manifestations. Fragility fractures leading to difficulty in mobility were seen in 13(12.6%) patients. Overall, 26(25%) patients suffered with depression and headache in 23(22.3%) of patients (Fig.1). Total 40(39%) patients had their DXA scan done out of which 31(30%) had osteoporosis and 06 (5.8%) had osteopenia. Vitamin D deficiency was found in 69(67%) patients. Ostieits fibrosa cystica was found in 04 (3.9%) patients. None of the patients had associated Multiple Endocrine Neoplasia (MEN) syndrome.

**Fig.2:**
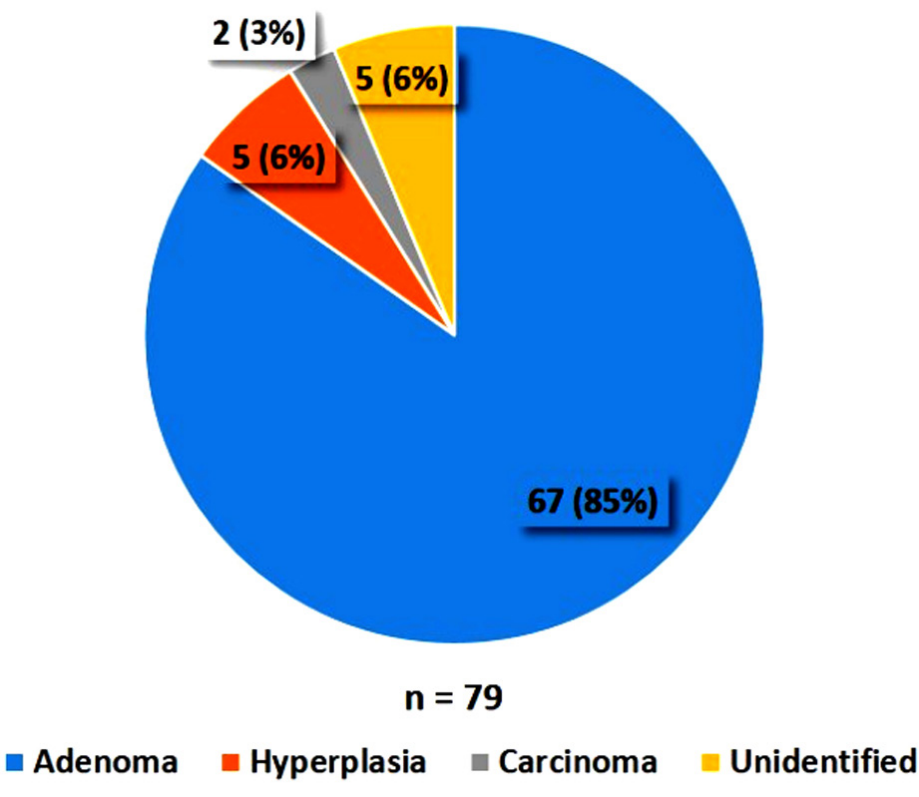
Histological distribution of patients with primary hyperparathyroidism n = Total number of Patients who underwent surgery.

Majority of the patients 79(76.7%) underwent surgical treatment whereas, one fourth of patients opted for medical treatment. Histology revealed that 67(85%) out of those 79 patients had a parathyroid adenoma, whereas hyperplasia was seen in 05(6%) and carcinoma in 02(3%) patients. Histology was not conclusive in 05(6%) out of the 79 patients who underwent surgery (refer to Fig.2). On kidney ultrasound, 30 (29.1%) patients had renal calculi and 01(0.98%) patient had nephrocalcinosiss. Ultrasound neck and sestamibi scan were able to localize the lesion in 66 (64.1%) and 77 (76.2%) patients respectively.

Mean serum calcium levels were improved post-surgery from 12.1 ± 1.8 mg/dl to 8.9 ± 0.8 mg/dl. Similarly, post-surgery PTH levels were decreased from 653.6 ± 683.4 pg/ml to 61.1 ± 88.7 pg/ml (Table-I). Post-operative hypocalcemia developed in 7 (8.8%) patients. Disease recurrence as manifested by elevation of PTH on follow up visit was seen in 13 (16.5%) out of the 79 patients who underwent surgery.

**Table-I:** Biochemical parameters of study participants.

	Normal Range	Means ± SD	Range (Min-Max)
Serum Calcium (mg/dl) (Pre-operative)	8.1 - 10.4	12.1 ± 1.8	8.8 - 18.8
Serum Calcium (mg/dl) (Post-operative)	8.1 - 10.4	8.9 ± 0.8	5.8 - 11.1
Serum PO4 (mg/dl)	2.5 - 4.8	2.4 ± 0.8	1 - 4.5
Serum PTH (pg/ml) (Pre-operative)	16 - 81	653.6 ± 683.4	76 – 2500
Serum PTH (pg/ml) (Post-operative)	16 - 81	61.1 ± 88.7	3 – 575
Serum Creatinine (mg/dl)	0.5 - 1.2	0.8 ± 0.3	0.3 - 1.9
Serum Albumin (g/dl)	3.5 - 5.2	3.8 ± 0.7	2 - 5.2
Vitamin D3 ng/ml	Sufficiency>30	23.4 ± 15.1	4 – 101

## DISCUSSION

The mean age of our patients at time of diagnosis was 59.3±16.2 years. Although this age is significantly higher from the mean age reported by Biyabani^8^ and Ahsan^9^ in earlier studies from Pakistan and India^4^, this is comparable to the findings of Yeh^11^ where the mean age of presentation in the Asian subgroup was 60.5 years. This may be due to the fact that majority of our patients were from an urban population of Karachi where nutritional status and socioeconomic conditions are generally better compared to other parts of the country therefore skeletal manifestations were probably delayed in this cohort. A striking finding in this study was the unexplained female predominance (80.6%). This has also been reported in other studies^4^,^9^,^12^ but any causal relationship between gender and the disease yet remains unknown.

In this series we have demonstrated that in Pakistan PHPT still presents with overt skeletal and renal disease. More than half of the patients (58.3%) sought medical attention because of some form of musculoskeletal complications such as bone pain, generalized body aches or proximal muscle weakness and 12.6% had a fragility fracture by the time of diagnosis. This degree of severity of skeletal disease in the Asian population affected by PHPT has consistently been observed across various studies from the region.^13^-^15^ A study from India showed that bone pains and painful proximal myopathy were the commonest presentation (47%), followed by pathological fractures in 23.5% cases.^13^ This percentage was even higher in an Iranian study where (93.5%) patients suffered from bone pains, deformities, pathologic fractures, and localized bone tumors.^15^ Ostietis fibrosa cystica (OFC), the classic form of skeletal complications of PHPT is now rarely seen in the developed countries. However, OFC still remains a predominant presentation of PHPT in this region.^7^,^8^,^16^ In our study, four patients (3.8%) had OFC. All of them had a concomitant Vitamin D deficiency. This extent of skeletal disease may partly be attributed to coexisting vitamin D deficiency as long standing Vitamin D deficiency can further exaggerate the secretion of PTH. Although we know that Vitamin D and calcium nutrition play a role in determination of severity of skeletal disease but It is yet not clear whether the austerity of skeletal disease in this region is only because of hypovitaminosis D and calcium malnutrition or there are any additional pathogenic environmental factors or genetic and racial differences playing a role. This hypothesis is further supported by a Chinese study which compared BMD in patients with PHPT in a single Centre from China to that of USA and noted lower bone mineral density in Chinese population as compared to USA in patients with PHPT.^14^ Same goes for the renal manifestations the rate of which is higher in Asian population as compared to the West. Ultrasound abdomen was done in all patients including those presenting with abdominal pain to look for renal calculi and nephrocalcinosis. Renal manifestations were found in 27.2% of our patients which is comparable to the findings reported in studies from our region.^4^,^8^-^10^ Further research is required to fully understand the racial, genetic and environmental factors involved if any and their potential effects on the clinical profile of patients in this part of world. Lipase and amylase were checked in 6(5.8%) patients due to suspicion of pancreatitis which all turned out to be within normal range. Overall, 26 (25%) patients reported having a preexisting diagnosis of depression at the first clinic visit with the endocrinologist. This high incidence of depression seen in this cohort, however, is comparable to the incidence of depression in the general population of Pakistan and a cause effect relationship with PHPT could not be established. It is interesting to note that 22% of the patients were asymptomatic at presentation and were referred to the endocrinology service upon incidental finding of hypercalcemia on their routine health checkup.

Normocalcemic hyperparathyroidism is a newer phenotypic variant of PHPT that has evolved over the last two decades as a result of the routine evaluation of serum biochemistry in the developed countries.^17^,^18^ Although the dynamics of this entity are not fully understood at the moment, various studies have shown that it is not an indolent condition and patients with normocalcemic hyperparathyroidism also have an increased prevalence of bone and renal complications.^19^-^21^ Although increasingly seen in modern day practice, the epidemiology of normocalcemic primary hyperparathyroidism is not exactly known,. In a population based study from Canada, the prevalence was 16.7%.^22^ In a study from Lahore, Pakistan, normocalcaemia was seen in 21.88% of patients.^10^ However, in our series, only one patient had normocalcemia. There was coexistent vitamin D deficiency in this patient and a history of fragility fracture. It is important to note that Vitamin D deficiency can cause calcium levels to fall in the normal range and the normocalcemia in this situation may not accurately reflect the gravity of disease as such patients may become hypercalcemic on correction of Vitamin D levels. There is also a possibility of missing the diagnosis if only the absence of hypercalcemia is used to exclude the diagnosis of PHPT. It is prudent for the clinicians to understand this interplay of Calcium, Vitamin D and PTH and that this may have influenced the disease presentation in our region. The clinicians should be vigilant to identify and correct Vitamin D deficiency in PHPT patients before making treatment decisions. It is also important to mention here the other end of the paradigm. In the recent years a trend has been seen among clinicians in Pakistan to empirically prescribe vitamin D to patients with musculoskeletal pains without documenting vitamin D deficiency. This has led to cases of Vitamin D intoxication and hypercalcemia. The situation can be critical if this practice is done in a patient of PHPT^23^ and implies that Vitamin D should be checked in all patients suspected of having PHPT and prescribed only if needed.

There is wide variability in the location of parathyroid gland and various noninvasive techniques have been developed for preoperative localization of disease in order to make the surgical procedure minimally invasive. The most widely used and credible modality is Technetium 99 sestamibi and has shown high sensitivity for pre-operative localization in PHPT.^24^ Accurate localization was done in 76.2% of our patients upon sestamibi scan. Nonetheless, Ultrasound of neck was also seen to be an effective localization tool as it is cheap and readily available in a cost constrained healthcare system and was able to localize the lesion in 64.1 % of our patients. Ectopic location of parathyroid gland in the mediastinum was detected in four cases.

Surgical excision remains the standard definitive treatment for PHPT. All patients were offered surgery as first line treatment, however, one fourth of patients refused surgery and opted for medical treatment. Majority of our patients underwent surgical treatment (76.7%). Excision of the involved gland was performed in cases where pre-operative localization was achieved whereas bilateral neck exploration was performed in the non-localized cases. However, recurrence of the disease was seen in 13(12.6%) patients. This may partly be attributed to the failure to localize the lesion on pre-operative imaging in nine patients and ectopic location of parathyroid glands in the mediastinum in two of them. Median sternotomy was done in cases of ectopically located parathyroid gland. Hungry bone syndrome occurred in seven of our patients post operatively and was managed without any complications. The low incidence of post-operative hypocalcemia and hungry bone syndrome was probably because of the practice in our Centre to replete Vitamin D in all patients who are deficient pre operatively. In the Western data, a significant cases of PHPT are associated with MEN syndrome,^25^ however there is no data from Pakistan regarding PHPT in context of MEN syndrome. To the best of our knowledge none of our patients had PHPT associated with MEN syndrome although genetic testing was not done. It is imperative to screen all patients presenting with PHPT at age younger than 40 years for MEN syndromes by genetic testing. The fact that genetic testing was not done in our patients who were 40 years of age or younger reflects the potential lack of awareness among clinicians about possibility of associated MEN syndromes in these patients. Clinicians need to understand the seriousness of this entity and need to be more vigilant not to miss the diagnosis.

### Limitation of this study

It was a single center study with small number of patients and most of them from an urban population therefore it may not reflect the actual picture of the entire Pakistani population, however it effectively highlights the morbidity associated with PHPT in our population.

The disease severity may partly appertain to the delay in seeking medical care due to illiteracy, lack of awareness and inaccessibility of healthcare facilities in a financially constrained population of this country. The conundrum if this difference in presentation and disease severity from the West is only a matter of nutritional and socioeconomic differences or there are any other genetic factors playing a role remains to be answered. Targeted measures like improving patient awareness, routine calcium screening, vitamin D supplementation and a high index of suspicion by the clinician may help in early diagnosis of the condition and thus reducing morbidity.

### Authors’ Contribution:

**TF:** Conceived, designed the study and did data collection, manuscript writing and it responsible for integrity of this study.

**BD:** Did data collection and entry.

**SS:** Did statistical analysis.

**NI:** Did review of manuscript.

## References

[1] Wermers RA, Khosla S, Atkinson EJ, Achenbach SJ, Oberg AL, Grant CS (2006). Incidence of primary hyperparathyroidism in Rochester, Minnesota 1993–2001:an update on the changing epidemiology of the disease. J Bone Mineral Res.

[2] Adami S, Marcocci C, Gatti D (2002). Epidemiology of primary hyperparathyroidism in Europe. Journal of bone and mineral research:Off J Am Soc Bone Mineral Res.

[3] Watts N, Bilezikian J, Camacho P, Greenspan S, Harris S, Hodgson S (2010). American Association of Clinical Endocrinologists Medical Guidelines for Clinical Practice for the diagnosis and treatment of postmenopausal osteoporosis. Endo Prac.

[4] Bhadada SK, Arya AK, Mukhopadhyay S, Khadgawat R, Sukumar S, Lodha S (2018). Primary hyperparathyroidism:insights from the Indian PHPT registry. J Bone Mineral Metabol.

[5] Bilezikian JP, Rubin M, Silverberg SJ (2006). Asymptomatic primary hyperparathyroidism. Arquivos Brasileiros de Endocrinologia Metabologia.

[6] Pradeep PV, Jayashree B, Mishra A, Mishra SK (2011). Systematic review of primary hyperparathyroidism in India:the past, present, and the future trends. Int J Endocrinol.

[7] Bhansali A, Masoodi SR, Reddy KS, Behera A, das Radotra B, Mittal BR (2005). Primary hyperparathyroidism in north India:a description of 52 cases. Ann Saudi Med.

[8] Biyabani SR, Talati JJ (1999). Bone and renal stone disease in patients operated for primary hyperparathyroidism in Pakistan:is the pattern of disease different from the west?. J Pak Med Assoc.

[9] Ahsan T, Erum U, Pal KM, Jabeen R, Qureeshi SG, Rehman UL (2017). The many guises of primary hyperparathyroidism:an unchanged scenario. J Pak Med Assoc.

[10] Afzal A, Gauhar TM, Butt WT, Khawaja AA, Azim KM (2011). Management of hyperparathyroidism:A live year surgical experience. J Pak Med Assoc.

[11] Yeh MW, Ituarte PH, Zhou HC, Nishimoto S, Amy Liu IL, Harari A (2013). Incidence and prevalence of primary hyperparathyroidism in a racially mixed population. J Clin Endocrinol Metabol.

[12] Mazeh H, Sippel RS, Chen H (2012). The role of gender in primary hyperparathyroidism:same disease, different presentation. Ann Surg Oncol.

[13] Muthukrishnan J, Jha S, Modi KD, Jha R, Kumar J, Verma A (2008). Symptomatic primary hyperparathyroidism:a retrospective analysis of fifty one cases from a single centre. J Assoc Physic India.

[14] Meng L, Liu S, Al-Dayyeni A, Sheng Z, Zhou Z, Wang X (2018). Comparison of initial clinical presentations between primary hyperparathyroidism patients from new brunswick and changsha. Int J Endocrinol.

[15] Bahrami A, Montazeri V, Barband AR, Pourzand A, Mobaseri M (2006). Advanced bone disease as the most common clinical presentation of primary hyperparathyroidism in Iranians:clinical and laboratory features of 62 patients from North-Western Iran. Int J Endocrinol Metab.

[16] Mishra SK, Agarwal G, Kar DK, Gupta SK, Mithal A, Rastad J (2001). Unique clinical characteristics of primary hyperparathyroidism in India. Br J Surg.

[17] Bilezikian JP, Khan AA, Potts JT (2009). Third international workshop on the management of asymptomatic primary hyperthyroidism. Guidelines for the management of asymptomatic primary hyperparathyroidism:Summary statement from the third international workshop. J Clin Endocrinol Metab.

[18] Cusano NE, Silverberg SJ, Bilezikian JP (2013). Normocalcemic primary hyperparathyroidism. J Clin Densitom.

[19] Marques TF, Vasconcelos R, Diniz E, Rego D, Griz L, Bandeira F (2011). Normocalcemic primary hyperparathyroidism in clinical practice:an indolent condition or a silent threat?. Arquivos Brasileiros De Endocrinologia Metabologia.

[20] Tordjman KM, Greenman Y, Osher E, Shenkerman G, Stern N (2004). Characterization of normocalcemic primary hyperparathyroidism. Am J Med.

[21] Charopoulos I, Tournis S, Trovas G, Raptou P, Kaldrymides P, Skarandavos G (2006). Effect of primary hyperparathyroidism on volumetric bone mineral density and bone geometry assessed by peripheral quantitative computed tomography in postmenopausal women. J Clin Endocrinol Metabol.

[22] Garcia-Martin A, Reyes-Garcia R, Munoz-Torres M (2012). Normocalcemic primary hyperparathyroidism:one-year follow-up in one hundred postmenopausal women. Endocrine.

[23] Bala S, Shah B, Rajput P, Rao P (2015). Unmasking of primary hyperparathyroidism by Vitamin D therapy. Ind J Nephrol.

[24] Moka D, Voth E, Dietlein M, Larena-Avellaneda A, Schicha H (2000). Technetium 99m-MIBI-SPECT:a highly sensitive diagnostic tool for localization of parathyroid adenomas. Surgery.

[25] Moline J, Eng C (2011). Multiple endocrine neoplasia type 2:An overview. Genet Med.

